# Tooth Anomaly, from Dr. S. P. Cutler

**Published:** 1876-06

**Authors:** 


### EDITORIAL, ETC.

---

*Tooth Anomaly.*—From S. P. Cutler, M. D., D. D. S., of Memphis, Tennessee, we have the following :

My friend, Dr. Mewlorn, of this place, extracted a left superior *dens sapientiæ,* for a man about 50 years old, preparatory for a set of teeth.

All the other teeth except the front ones had been out several months. It was after the healing of the gums that the outer cusp of the tooth showed itself.

On extraction, a portion of the enamel crushed, it being but a mere shell, though not external decay.

The fang very much exostosed from apex up to and over the edge of enamel half way around, with abrupt offset.

The doctor noticing something strange about it, washed it and examined into it. Instead of a common decay under the portion where the enamel gave away, there was a fleshy mass, completely filling the hollow, white, smooth, and elastic, which he removed ; it was slightly adherent at bottom. When he showed it to me next day, the soft mass had dried and swiveled up very much, which on placing in glycerine, filled out full and plump, nearly the size of the cavity. The cavity occupies the inner anterior portion of the crown down as low as enamel.

When extracted the enamel was perfect, but crushed in on one side; there was no external opening, on replacing the broken enamel, the tooth externally was perfect. This excavation, as I will call it, extended down nearly to the pulp, showing the exact shape of upper surface with a very thin stratum of bone over the pulp, white, sound, and no discoloration anywhere; having a frosted appearance. Before the enamel crushed, this tooth would have been taken as a perfectly sound one with no outward sign of disease under this thin stratum of dentine, the pulp was normal, though down the fang it was ossified to apex, more or less. The above are the descriptive features of the case.

Microscopic appearances of the soft mass: I sliced the soft mass after treating in glycerine, with a razor into thin sections for examination. The slices are composed chiefly of connective tissue fibres, arranged as lineal sells, beautifully clear and distinct, running parrallel. Some few patches of true dental nerve fibrils were visible.

Some darker spots, were filled with beautiful triple phosphate crystals, of large size, many in numbers, in the bottom portion, adjacent to pulp. On treating a section with liquor potassa, the crystals become more distinct and detached from the stroma of connective tissue. There where other crystals, long prismo and large platis in small quantities.

Why the triple phosphate crystals of ammonia and magnesia should be found here in this softened dentine, is certainly a great mystery of no ordinary character, as they are generally found only in urinary deposits, of certain conditions of the kidneys. If these crystals had been of the triple phosphate of lime, there would not be so much obscurity in the case. The thin stratum of uniform thickness, over the surface of pulp, contained myriads of tubular openings in normal condition, no discoloration; the pulp underneath, though dried up when examined, gave the usual pulp structures, and from what could be seen under the microscope, there can be no doubt, about the vital fibrillar connection, just the same as before the lime was absorbed away, and nature may in time, had the tooth remained in, have restored the best lime; why not? There can be no doubt about this soft mass having been once hard and firm, like the balance. The removal of the lime, from internal absorption leaving the connective tissue stroma, and nerve fibrils, all in thin normal position, obscures the tubular structure, owing to minuteness of the mere fibrils, running as they do, parallel with the other fibris. The safe structure above referred to, is no doubt true and exact structure of dentine, minus the lime. This structure then, may be regarded as the true dentinal matrix, that is formed successively, just prior to dentification, or rather in advance of ossification. It is well known that there are no blood vessels of any kind in dentine, only asmosic action, exas and endasmose, of liquor sanguinis, to nourish the fibrils after the dentine is once found.

No specimen treated with acids for the removal of the lime in dentine, or where the lime has been removed from decay, ever retains the exact normal structure, as seen in the specimen. The question is what process removed the lime from *the* solid firm dentine? Could there have been an acid diathesis in *loco*, that removed the lime by liquifaction, then absorption through the tubuli into the pulp curculation? Who will salve this problem? There were no parasites in the case of any kind.

---

